# Validating wearable step counts in multiple sclerosis research: a replication study

**DOI:** 10.3389/fneur.2025.1709389

**Published:** 2025-10-30

**Authors:** Myla D. Goldman, Shanshan Chen, Bhavana Kunisetty, Jeffrey M. Gelfand, Bruce A. C. Cree, Valerie J. Block

**Affiliations:** ^1^Department of Neurology, Virginia Commonwealth University School of Medicine, Richmond, VA, United States; ^2^Department of Biostatistics, School of Public Health, Virginia Commonwealth University, Richmond, VA, United States; ^3^Division of Neuroimmunology and Glial Biology, Department of Neurology, Weill Institute for Neurosciences, University of California San Francisco, San Francisco, CA, United States; ^4^Department of Physical Therapy and Rehabilitation Science, University of California, San Francisco, San Francisco, CA, United States

**Keywords:** Expanded Disability Status Scale [EDSS], wearables, multiple sclerosis, activity monitoring, accelerometer

## Abstract

**Introduction:**

For people with multiple sclerosis (MS), mobility impairment is common and a significant contributor to reduced quality of life. With advancements in wearable technology, step count data has emerged as a promising method to track mobility and monitor functional decline. However, studies comparing the replicability of wearable mobility data using varying devices remain limited in MS populations.

**Methods:**

This study investigates the reliability of step count data and its associations with clinical outcomes in MS patients using two independent cohorts with different wearable devices: California (CA) (*n* = 97 Fitbit wrist sensor, 4-week wear) and Virginia (VA) (*n* = 61; ActiGraph hip sensor, 7-day wear). We analyzed correlations between average daily step counts and common MS clinical measures [disability: Expanded Disability Status Scale (EDSS); walking speed: Timed 25-Foot Walk (T25FW)] as well as patient-reported outcomes (12-item MS walking scale, MSWS-12, Modified Fatigue Impact score, MFIS).

**Results:**

Analysis of the VA cohort revealed similar average daily step counts to those seen in the CA cohort (6,010 vs. 5,478 steps/day). Step count variability (standard deviation) decreased with increasing EDSS in both cohorts. Step counts in the VA cohort were significantly correlated with EDSS (*r* = −0.34), T25FW (*r* = −0.58), MSWS-12 (*r* = −0.57), and MFIS (*r* = −0.45), similar to findings from the CA cohort. Additionally, within-subject reliability over 7 days was moderate (ICC = 0.599), with high correlations between 4-day and 7-day averages (*r* ≥ 0.98).

**Discussion:**

The step count analyses from two different wearable devices show replicable associations with clinical and patient-reported outcomes in MS, highlighting their promise as digital biomarkers for clinical monitoring and care, rehabilitation, and patient self-management.

## Introduction

Multiple Sclerosis (MS) is an autoimmune disorder of the central nervous system that results in multi-faceted neurologic impairment and loss of function (1). Although many domains are impacted by MS, among the most common and significant for patients are limitations in mobility (2). These mobility changes can significantly impact a person with MS’s quality of life, independence, and ability to participate in daily activities (3). Improved understanding and validation of outcome measures focused on mobility in MS are crucial for optimization of clinical management, targeted interventions, and supporting patient self-management and education. Validated in-clinic outcome measures are commonly applied in clinical research, but there has been growing interest in and application of wearable technology as a tool to understand the real-world impact of walking impairment in MS beyond in-clinic assessments (4–7). These forms of data provide information beyond the structured clinical environment and enhance our understanding of the day-to-day impact of MS, and have the potential to serve as early indicators of change in a patient’s disability status. Despite their growing use, validation of wearable devices in people with MS (pwMS) remains limited, particularly in examining whether findings are replicable across different devices and geographically separate cohorts.

In MS, and other neurological disorders, research has established that the step count metrics from remote wearable activity monitoring devices are associated with disability levels [as measured by the Expanded Disability Status Scale (EDSS)], walking speed [via the Timed 25-Foot Walk (T25FW)] and patient-reported disease impact outcomes [e.g., Multiple Sclerosis Walking Scale (MSWS-12)] (8–10). These objective measurements offer potential early indicators of functional decline, enabling more timely and personalized interventions. In 2019, we published a foundational study to demonstrate the utility of wearable metrics in a California-based cohort [CA], using data from wrist-worn Fitbit devices to characterize step count patterns in MS patients (11, 12). The findings showed that daily step counts were significantly correlated with EDSS, T25FW, MSWS-12, and fatigue severity. However, independent replication of these findings in different patient populations and with a broader range of devices remained a research gap.

To address this gap, we aimed to replicate and validate these findings through secondary analysis of an independent cohort from a different geographic location, employing a different wearable device (ActiGraph GT3-X accelerometer) in a Virginia-based cohort [VA] (13). In comparing step count metrics and their relationship to clinical and patient-reported outcome measures across cohorts with differing characteristics, devices, and data collection protocols, this study aimed to assess the generalizability and robustness of step count data as a valuable tool for supporting clinical management, rehabilitation, and education/self-management in MS.

## Methods

We obtained the original activity data from the CA (11, 12) and VA (13) cohorts, harmonized overlapping variables collected in both studies, and conducted secondary data analyses to replicate analyses in the CA cohort using the VA dataset (13). For detailed descriptions of data collection procedures, see the CA (11, 12) and VA (13) published studies. Below, we provide a brief overview of the two study protocols and variables analyzed.

### California (CA) cohort

A year-long study that recruited 99 relapsing or progressive MS patients from the University of California, San Francisco (UCSF) MS Center between July 2015 and April 2016. Recruitment was stratified by EDSS had a target range of EDSS 0–6.5. Clinical and patient-reported outcome (PRO) measures included EDSS, the MSWS-12, the T25FW, and the 5-item Modified Fatigue Impact Scale (MFIS-5). At study entry, participants were instructed to wear a Fitbit flex accelerometer on their nondominant wrist as much as possible for 4 weeks except while swimming. Participants were trained on set-up and management (recharging the device every 5–8 days) of the devices.

### Virginia (VA) cohort

A 2-year longitudinal study that recruited 62 MS and 40 healthy controls at the University of Virginia (2010–2015). MS participants were recruited from the Neurology outpatient clinic and had a confirmed diagnosis of either relapsing or progressive MS (13). Each subject completed five visits, at which clinical, PRO and physical activity data were collected. Overlapping measures with CA cohort, included EDSS, MSWS-12, T25FW, and MFIS. At each visit, participants wore the ActiGraph GT3X accelerometer; (ActiGraph, FL, United States) on their non-dominant hip for 7 consecutive days while awake (excluding swimming or bathing). Compliance was defined as ≥10 h/day for ≥3 valid days of weartime. Daily step counts were calculated by actigraphy data using ActiLife. For replication purposes, only baseline visit data of MS participants were included; data from healthy controls and follow-up visit were excluded.

### Statistical analysis

First, we compared demographic and EDSS distributions across cohorts using descriptive statistics. Next, we analyzed the step counts from the VA’s ActiGraph dataset. Daily activity counts <100 were considered unreliable (e.g., due to non-wear) and excluded from analysis. Following analyses from the CA cohort, we visualized:

1. Step counts by EDSS category,
2. Average daily step counts across all subjects,
3. Pearson correlations between the average daily step counts of *n* consecutive days (*n* = 1,2,3,…,6) and the 7-day averages.

We also estimated the intraclass correlation coefficients (ICC) with a 95% confidence interval (CI) to determine within-subject test–retest reliability of the repeated measures during the 7-day in-home data collection. Lastly, Spearman correlations were calculated among pairs of the following variables: age, body-mass index (BMI), disease duration since symptom onset, disease duration since diagnosis, EDSS, Paced Auditory Serial Addition Test (PASAT), T25FW, Modified Fatigue Impact Scale Total (MFIS Total), MSWS-12 Total, and daily step counts. Unlike the CA cohort, which assessed step counts using both Fitbit and ActiGraph devices, the VA cohort used only ActiGraph. Therefore, Bland–Altman analyses comparing the agreement of two devices were not analyzed. All analyses were conducted in R Studio (R version 4.4.3; R Studio Inc., Boston, Massachusetts).

## Results

### EDSS and Step Counts

A total of 61 of the MS participants in the VA cohort had an EDSS score and step count data and the distribution is shown in Table 1. Overall, this cohort had relatively lower disability measured by EDSS (aligned with the inclusion criteria), with one participant having the highest EDSS of 4.5. This is different from the CA cohort (97 participants with MS had both EDSS and step count data), where 48% of the cohort had an EDSS no less than 4.5, indicating that they had more MS related neurologic disability.

**Table 1:** Step count by cohort and disability.

	EDSS	/	1	1.5	2	2.5	3	3.5	4	4.5	/	/	/	/
VA cohort	No. of subjects	/	2	8	22	5	11	8	4	1	/	/	/	/
	No. of subjects	10			46				5					

	EDSS	0	1	1.5	2	2.5	3	3.5	4	4.5	5	5.5	6	6.5
CA cohort	No. of subjects	8	4	2	9	6	3	6	14	4	4	4	19	14
	No. of subjects	14			24				14	12			19	14

The average daily step count in the VA cohort was 6,010 steps per day, which is similar to the average daily step count in the CA cohort (∼5,478 steps per day), and is consistent with previous studies in MS overall (14–16).

**Figure 1:**
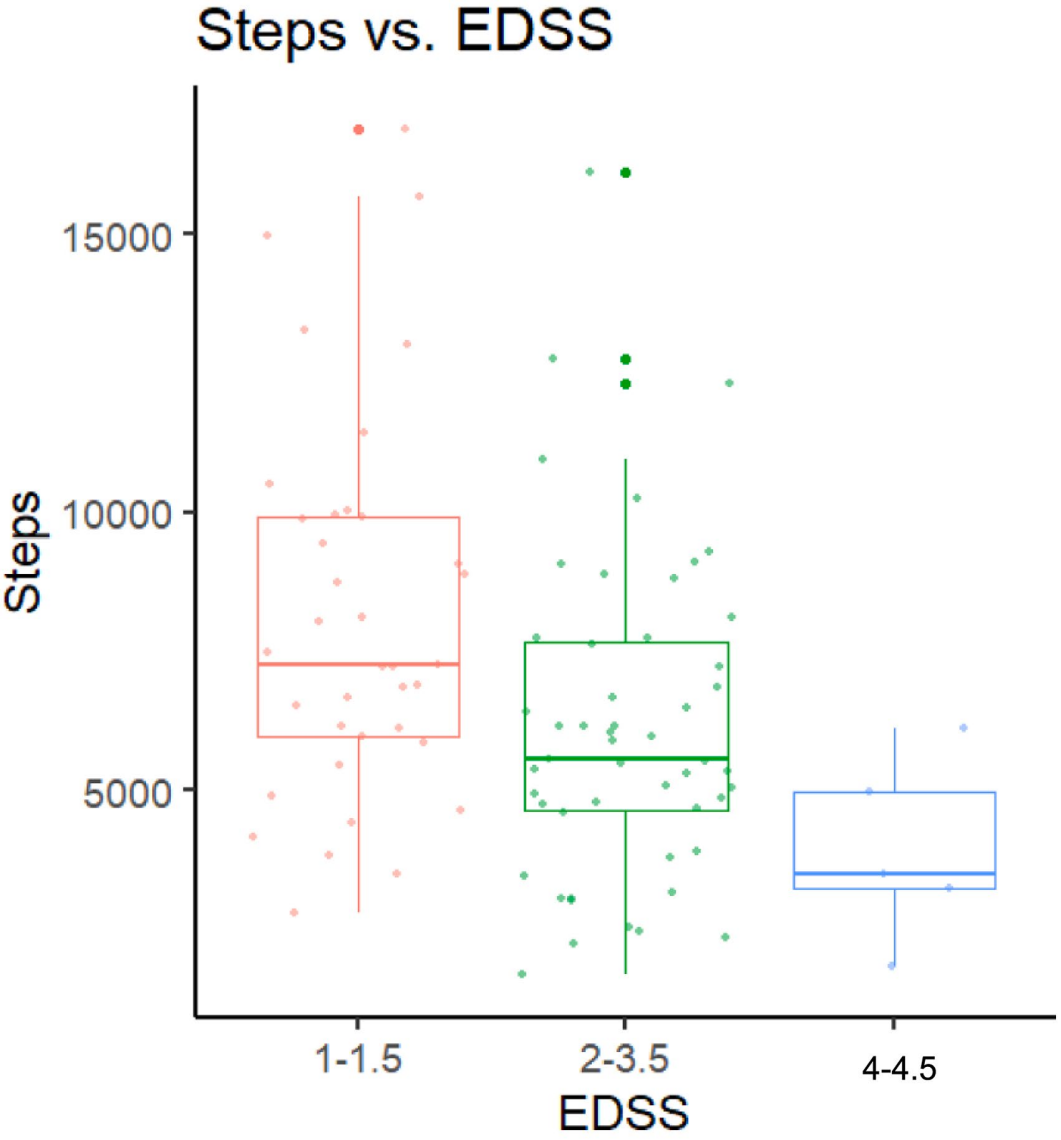
Average daily step counts for each EDSS group in the VA cohort.

In the VA cohort, step count variability was greatest in the lower EDSS range (0–1.5; SD 2,900 steps, range 4,910–15,000) and lowest in the EDSS 3.5–4.5 group (SD 1,650 steps, range 1,830–6,110). This pattern is consistent with the findings from CA cohort, which observed a wider step count range among participants with lower disability (EDSS: 0–1.5; 2,286–18,648 steps per day) and decreasing variability with higher EDSS scores. However, the VA cohort included only 5 participants in the higher EDSS range (4.0–4.5), limiting interpretation of variability in the higher disability (EDSS) groups (see Figure 1; Table 2).

**Table 2:** Average step count by disability group; VA cohort.

Steps	EDSS 0.0–1.5 (*N* = 10)	EDSS 2.0–3.5 (*N* = 46)	EDSS 4.0–4.5 (*N* = 5)	Overall (*N* = 61)
Mean (SD)	8,070 (2,900)	5,770 (2,690)	3,920 (1,650)	6,010 (2,830)
Median [Min, Max]	7,370 [4,910, 15,000]	5,480 [1,670, 16,100]	3,510 [1,830, 6,110]	5,540 [1,670, 16,100]
Missing	0 (0%)	3 (6.5%)	0 (0%)	3 (4.9%)

#### The association between EDSS and Daily Steps

**Figure 2:**
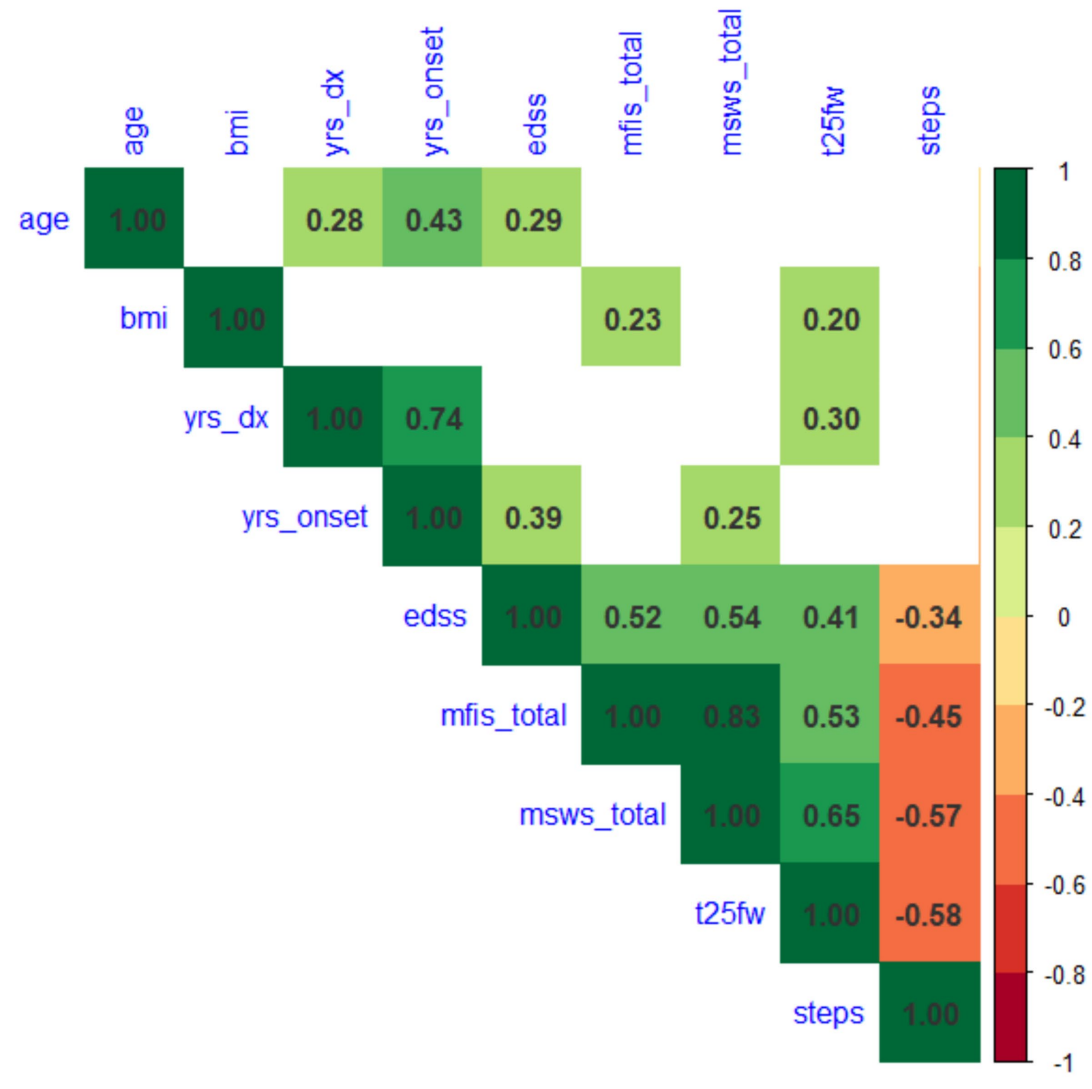
Spearman correlations between demographics, clinical outcomes (EDSS and T25FW), patient-reported outcomes (MFIS and MSWS-12) and daily step counts.

The spearman correlation between average daily steps and EDSS was lower in VA, (*r* = −0.34, *p* = 0.005; Figure 2) compared with CA (*r* = −0.71, *p* < 0.001).

### Reliability of 7-day Step Counts

**Figure 3:**
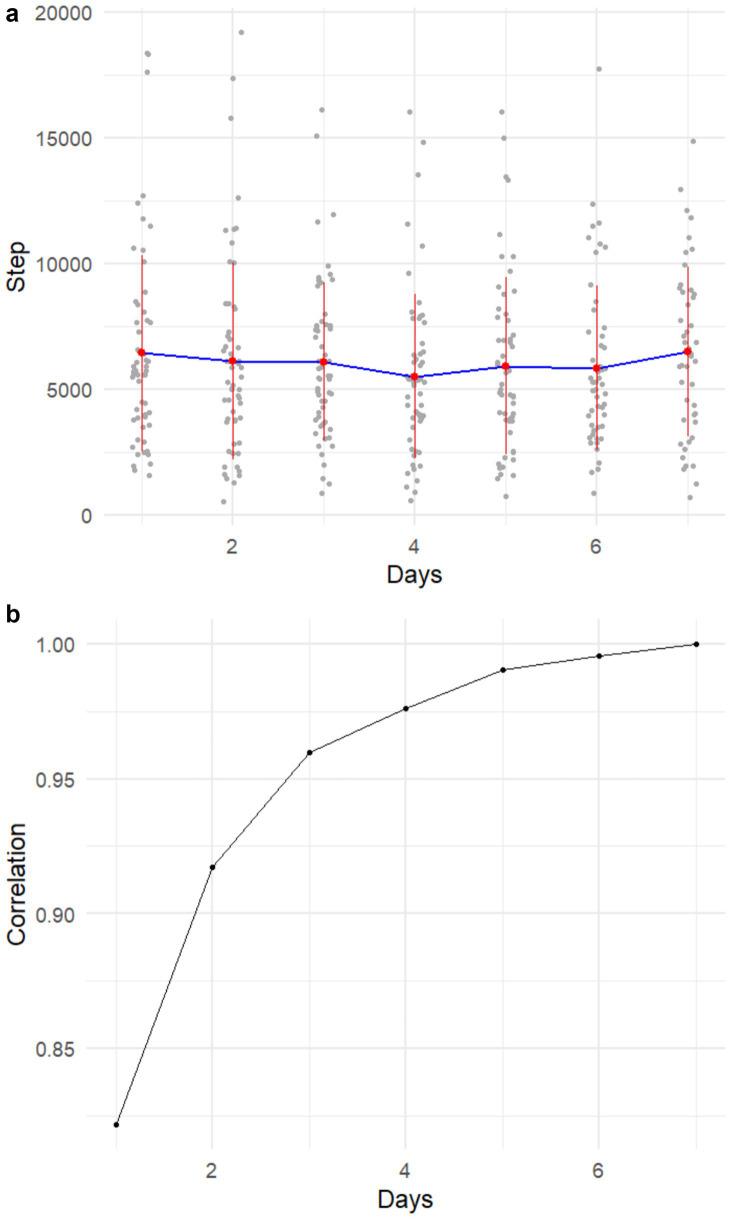
**(a)** Average daily step counts per day across all subjects. **(b)** Pearson correlations between the average daily step count of successive days and the average step count of the 7-day period.

In VA study, the within-subject ICC of daily steps was 0.60 (95% CI: 0.48 ~ 0.69). Similar to the CA study, we did not observe trends of reactivity within the 7-day data collection (Figure 3a). We also computed Pearson correlations of the average daily step count of *n* consecutive days and the average step count of 7 days (Figure 3b). Average step counts from 4 days correlated strongly with 7-day data (*r* ≈ 0.98), increasing to >0.99 with 5 days. Thus, while 7-day actigraphy remains standard, ≥4 days of data seem to provide reliable estimates in MS.

### Spearman correlations

Spearman correlations between MSFC, EDSS and steps in VA cohort are shown in Figure 2, with blank cells indicating the correlations are not significant (*p* > 0.05). Steps are significantly associated with walking outcomes with strongest correlation with T25FW (*r* = −0.58, *p* < 0.0001), followed by MSWS (*r* = −0.57, *p* < 0.0001), and MFIS (*r* = −0.45, *p* < 0.0001). This is comparable to the CA cohort findings: using Spearman’s correlations, wherein average daily step counts were strongly associated with T25FW (*r* = −0.65, *p* < 0.001), MSWS scores (*r* = −0.65, *p* < 0.001) and MFIS (*r* = −0.41, *p* < 0.01).

## Discussion

We investigated the replicability of step count measures across two geographically distinct MS cohorts using different activity monitoring devices. Mirroring the approach used in the CA cohort (11, 12) study of 97 participants with MS using wrist-worn Fitbit activity monitors, we replicated key findings in the VA cohort (13) of 61 participants with MS with less severe disability, assessed using the hip-worn ActiGraph sensors. Despite the differences in device type, wear location, and data collection site, both cohorts showed comparable mean step counts and variability across the comparable EDSS groups. We also demonstrated step counts demonstrated consistent and significant correlations to traditional clinic-based (i.e., EDSS, T25FW) and patient-reported (i.e., MSWS-12, MFIS) outcome measures.

Both studies demonstrated that at lower EDSS levels, the range and between-subject variance of step counts are larger than those at higher EDSS levels, suggesting the impact of MS on real-world mobility varies more among those with less mobility impairment due to MS. This is likely related to the disassociation between capacity and behavior, where behavior becomes a more relevant factor among those with greater capacity (17). This higher variance in step counts among participants with lower EDSS likely contributed to the weaker correlation between EDSS and step counts observed in VA cohort (*r* = −0.34) compared with the CA cohort (*r* = 0.56). As the VA cohort included a greater proportion of participants with less severe disability, step counts were more heterogeneous, reducing the strength of the association. Similarly, the lower within-subject reliability in VA cohort (ICC = 0.59, vs. 0.74 in CA cohort) may also stem from this step count variability at lower EDSS levels.

In both studies, step count correlated significantly with T25FW, MSWS-12, and MFIS at comparable levels (0.4–0.6). Among these, the strongest association was with T25FW (*r* = −0.58 in VA cohort, *r* = −0.65 in CA cohort), indicating that clinic-based walking speed reflects real-world mobility. Step counts were also strongly correlated with patient-reported walking ability (MSWS-12), though the correlation was slightly weaker in VA cohort (*r* = −0.57 vs. –0.65). Patient reported fatigue as measured by the MFIS, was comparably associated with step counts (*r* = −0.45 in VA cohort, and *r* = −0.44 in CA cohort), suggesting that fatigue experienced by pwMS impacts real-world mobility. From a clinical perspective, the results highlight the potential to establish thresholds of meaningful change in daily activity, facilitating interpretation of longitudinal changes and guiding individualized rehabilitation strategies.

This study had several limitations. First, as a secondary data analysis, the VA cohort did not match the CA cohort in terms of disability range (EDSS levels). The higher proportion of participants with lower EDSS in the VA cohort increased variability in step counts. Such heterogeneity across cohorts may introduce bias, influencing generalizability of the study. In addition, a few analyses could not be tested for replication due to unavailable variables, i.e., only the VA collected 9-Hole peg test, Symbol Digit Modalities Test, and only CA cohort collected patient reported bladder, bowel disability surveys and the Timed-Up and Go; mobility and balance test. Future studies should ensure that such measures (i.e., upper extremity function) are harmonized across cohorts so that replication of findings, including those observed here, can be evaluated across the full spectrum of disability, strengthening generalizability. Second, activity monitoring in the VA cohort followed the standard 7-day protocol, rather than the 4-week collection used by the CA cohort, potentially limiting the ability to capture longer-term activity patterns contributing to the lower observed ICC. Still, the CA cohort observed no reactivity and found that monitoring durations of about 2 weeks yielded stable results, suggesting limited impact of the shorter protocol. Thirdly, device placement differed (Fitbit wrist, ActiGraph waist), which may influence results; however, the CA cohort’s comparison over a 2-min walk and 7-day home monitoring showed no systematic bias between devices (Bland–Altman analysis). Despite these limitations, we believe comparison of these different cohorts provides valuable information regarding the replicability and application of wearable devices in MS populations.

In summary, this study demonstrates that step count measures are replicable across two geographically distinct MS cohorts despite differences in device type, wear location, and study design. By confirming consistent associations with both clinical and patient-reported outcomes, our findings strengthen the validity of wearable-derived mobility metrics in MS. These results support the use of digital mobility data as a practical tool for monitoring disease impact and advancing patient-centered care.

## Data Availability

The original contributions presented in the study are included in the article/supplementary material, further inquiries can be directed to the corresponding authors.

## References

[1] Cree BAC Hauser SL. Multiple Sclerosis In: Longo D Fauci A Kasper D Hauser S Jameson JL Loscalzo J, editors. Harrison's principles of internal medicine. 22nd ed. New York, NY: Mcgraw Hill (2026)

[2] Larocca NG. Impact of walking impairment in multiple sclerosis: perspectives of patients and care partners. Patient. (2011) 4:189–201. doi: 10.2165/11591150-000000000-00000, PMID: 21766914

[3] Prigent G Aminian K Gonzenbach RR April R Paraschiv-Ionescu A. Effects of multidisciplinary inpatient rehabilitation on everyday life physical activity and gait in patients with multiple sclerosis. J Neuroeng Rehabil. (2024) 21:88. doi: 10.1186/s12984-024-01383-0, PMID: 38807215 PMC11131212

[4] Sparaco M Lavorgna L Conforti R Tedeschi G Bonavita S. The role of wearable devices in multiple sclerosis. Mult Scler Int. (2018) 2018:7627643. doi: 10.1155/2018/7627643, PMID: 30405913 PMC6199873

[5] Zheng P Motl RW. Psychometric properties of free-living step-based metrics (daily steps and peak cadence) in multiple sclerosis. Arch Phys Med Rehabil. (2025):S0003-9993(25)00708-7. doi: 10.1016/j.apmr.2025.05.005 40398529

[6] Frechette ML Meyer BM Tulipani LJ Gurchiek RD Mcginnis RS Sosnoff JJ. Next steps in wearable technology and community ambulation in multiple sclerosis. Curr Neurol Neurosci Rep. (2019) 19:80. doi: 10.1007/s11910-019-0997-9, PMID: 31485896

[7] Woelfle T Bourguignon L Lorscheider J Kappos L Naegelin Y Jutzeler CR. Wearable sensor technologies to assess motor functions in people with multiple sclerosis: systematic scoping review and perspective. J Med Internet Res. (2023) 25:E44428. doi: 10.2196/44428, PMID: 37498655 PMC10415952

[8] Koch MW Mostert JP Wolinsky JS Lublin FD Uitdehaag B Cutter GR. Comparison of the EDSS, timed 25-foot walk, and the 9-hole peg test as clinical trial outcomes in relapsing-remitting multiple sclerosis. Neurology. (2021) 97:E1560–70. doi: 10.1212/WNL.0000000000012690, PMID: 34433679 PMC9246083

[9] Goldman MD Ward MD Motl RW Jones DE Pula JH Cadavid D. Identification and validation of clinically meaningful benchmarks in the 12-item multiple sclerosis walking scale. Mult Scler. (2017) 23:1405–14. doi: 10.1177/1352458516680749 27903937 PMC5411321

[10] Kalinowski A Cutter G Bozinov N Hinman JA Hittle M Motl R. The timed 25-foot walk in a large cohort of multiple sclerosis patients. Mult Scler. (2022) 28:289–99. doi: 10.1177/13524585211017013, PMID: 34100297 PMC8795230

[11] Block VJ Bove R Zhao C Garcha P Graves J Romeo A. Association of continuous assessment of step count by remote monitoring with disability progression among adults with multiple sclerosis. JAMA Netw Open. (2019) 2:E190570. doi: 10.1001/jamanetworkopen.2019.0570, PMID: 30874777 PMC6484622

[12] Block VJ Lizee A Crabtree-Hartman E Bevan C Graves J Bove R. Continuous daily assessment of multiple sclerosis disability using remote step count monitoring. J Neurol. (2016) 88:1–11. doi: 10.1212/WNL.88.16_supplement.P1.376, PMID: 27896433 PMC5292081

[13] Goldman MD Chen S Motl R Pearsall R Oh U Brenton JN. Progression risk stratification with six-minute walk gait speed trajectory in multiple sclerosis. Front Neurol. (2023) 14:1259413. doi: 10.3389/fneur.2023.1259413, PMID: 37859654 PMC10582752

[14] Dlugonski D Pilutti LA Sandroff BM Suh Y Balantrapu S Motl RW. Steps per day among persons with multiple sclerosis: variation by demographic, clinical, and device characteristics. Arch Phys Med Rehabil. (2013) 94:1534–9. doi: 10.1016/j.apmr.2012.12.014, PMID: 23419331

[15] Cavanaugh JT Gappmaier VO Dibble LE Gappmaier E. Ambulatory activity in individuals with multiple sclerosis. J Neurol Phys Ther. (2011) 35:26–33. doi: 10.1097/NPT.0b013e3182097190, PMID: 21475081

[16] Arntzen EC Bidhendi-Yarandi R Sivertsen M Knutsen K Dahl S Hartvedt M. The effect of exercise and physical activity-interventions on step count and intensity level in individuals with multiple sclerosis: a systematic review and meta-analysis of randomized controlled trials. Front Sports Act Living. (2023) 5:1162278. doi: 10.3389/fspor.2023.1162278, PMID: 37583464 PMC10425270

[17] Engelhard MM Patek SD Lach JC Goldman MD. Real-world walking in multiple sclerosis: separating capacity from behavior. Gait Posture. (2018) 59:211–6. doi: 10.1016/j.gaitpost.2017.10.015, PMID: 29078135 PMC5695705

